# Computational Optimization of the Size of Gold Nanorods for Single-Molecule Plasmonic Biosensors Operating in Scattering and Absorption Modes

**DOI:** 10.1021/acs.jpcc.1c02510

**Published:** 2021-07-01

**Authors:** Teresa Staniszewska, Maciej Szkulmowski, Seweryn Morawiec

**Affiliations:** Institute of Physics, Faculty of Physics, Astronomy and Informatics, Nicolaus Copernicus University in Torun, Grudziadzka 5, 87-100 Torun, Poland

## Abstract

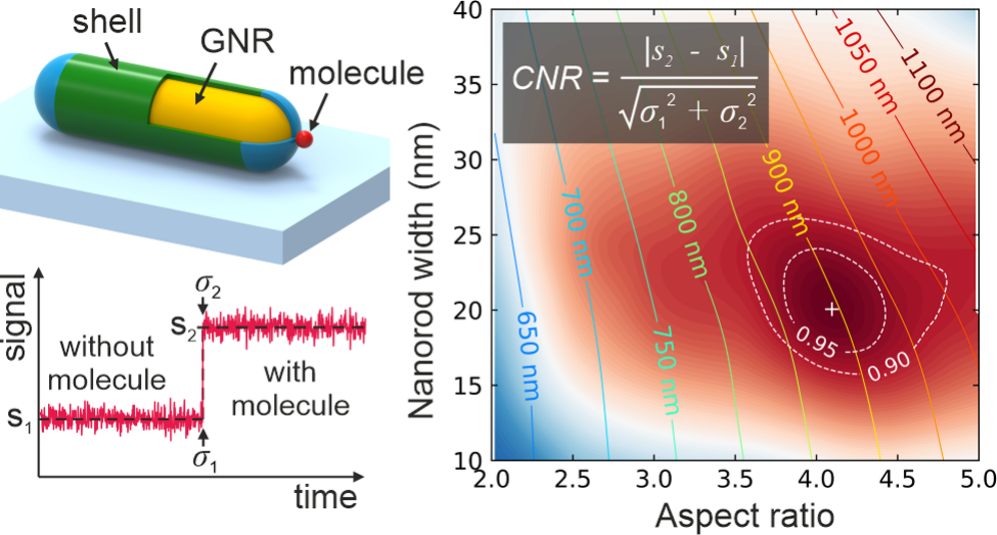

We present a comprehensive computational study on the optimization of the size of gold nanorods for single-molecule plasmonic sensing in terms of optical refractive index sensitivity. We construct an experimentally relevant model of single-molecule–single-nanoparticle sensor based on spherically capped gold nanorods, tip-specific functionalization and passivation layers, and biotin-streptavidin affinity system. We introduce a universal figure of merit for the sensitivity, termed contrast-to-noise ratio (CNR), which relates the change of measurable signal caused by the discrete molecule binding events to the inherent measurement noise. We investigate three distinct sensing modalities relying on direct spectral measurements, monitoring of scattering intensity at fixed wavelength and photothermal effect. By considering a shot-noise-limited performance of an experimental setup, we demonstrate the existence of an optimum nanorod size providing the highest sensitivity for each sensing technique. The optimization at constant illumination intensity (i.e., low-power applications) yields similar values of approximately 20 × 80 nm^2^ for each considered sensing technique. Second, we investigate the impact of geometrical and material parameters of the molecule and the functionalization layer on the sensitivity. Finally, we discuss the variable illumination intensity for each nanorod size with the steady-state temperature increase as its limiting factor (i.e., high-power applications).

## 1 Introduction

Noble-metal nanoparticles sustaining plasmonic resonances are perfectly suited to detect and study single organic molecules with no need for fluorescent labeling.^1−3^ The detection of biomolecules is facilitated by pronounced spectral shifts of the resonance induced by the binding of an analyte molecule to the receptor molecule stabilized on the nanoparticle’s surface.

The resonant optical response of metallic nanoparticles, i.e., localized surface plasmon resonance (LSPR), arises from the coupling of light to the collective oscillations of free electrons confined within a nanoparticle. In turn, the collective oscillations produce strong enhancements of the near optical field and give rise to absorption and scattering in the optical far field.^4^ The magnitude and spatial distribution of the near optical field; hence, the plasmonic response of a nanoparticle, depends strongly on its size, shape, material, and the refractive index of its environment. Notably, the magnitude of resonance shift induced by the molecular binding, representing the strength of interaction between the molecule and the plasmon mode, scales with the overlap integral between the molecule and the particle’s near-field enhancement.^5−7^ Therefore, the geometry of the nanoparticles and their surrounding need to be carefully optimized for the highest sensing performance.^8^

Among various geometries of metallic nanoparticles suitable for biosensing applications,^9^ gold nanorods (GNRs) are commonly employed^10−13^ as their longitudinal LSPR can be easily tuned across the visible and near-infrared wavelength range by varying their aspect ratio. Their elongated shape also red-shifts the resonance away from the interband transition reducing plasmon damping and increasing the near-field enhancements.^14^ GNRs can be synthesized by wet chemistry methods providing high-quality single-crystal nanoparticles and good control over the size and shape monodispersity.^15^ Gold is the usual material of choice due to its chemical stability and better control of synthesis compared with silver, despite the latter being able to provide stronger plasmonic response.^16^ Importantly, the existing chemical protocols allow for selective functionalization of highly curved surfaces of nanoparticles, i.e., the tips of the nanorod and passivation of remaining surfaces. This method restricts the possible binding sites to the areas with high local field enhancements^17^ enabling high sensitivity and specificity of single-molecule plasmonic biosensors.

Single-molecule plasmonic sensing relies on high signal-to-noise detection of individual plasmonic nanoparticles through either scattering or absorption.^3^ This requires high optical resolution and high-contrast imaging, which are usually achieved by background-free microscopy techniques. In the past years, label-free detection of single organic molecules has been successfully demonstrated by three distinct background-free detection techniques: (1) directly monitoring the resonance position by spectroscopic measurements using broad-band illumination and dark-field microscopy,^18^ (2) monitoring the scattering intensity at a fixed wavelength in total internal reflection microscopy,^19^ and (3) monitoring the absorption intensity at fixed wavelength via the photothermal effect.^20^ In each technique, the discrete binding events were detected as step functions in the signal registered over time. Importantly, in each case, the detection was hindered by extensive measurement noise.

The general goal for future biosensors is to detect the smallest organic molecules^21^ (i.e., below 50 kDa) that produce tiny resonance shifts, which are easily obscured by noise. Achieving this goal will rely on two essential factors, first, high signal-to-noise detection of single particles, and second, precise measurement of the LSPR shift. Thus, the crucial point is to optimize the geometry of the sensing particles, according to the sensing modality, for the highest sensitivity, i.e., the measurable signal in relation to the inherent measurement noise.

The problem of optimum nanorod size has been addressed in the literature; however, the existing data suffer from at least one of the following drawbacks: (1) do not consider the measurement noise, (2) do not exploit full parameter space by focusing on the aspect ratio only, or (3) consider bulk sensitivity rather than the single-molecule detection. Summarizing the published results, Becker et al.^22^ reported the optimum aspect ratio of GNRs between 3 and 4 for fixed nanorod width (20 nm) for both spectral and fixed-wavelength sensing using standard noise-independent figures of merit (FOMs). Nusz et al.^23^ discussed details of noise for spectral sensing and reported the optimum length between 55 and 65 nm and diameter between 25 and 35 nm for bulk/many-molecule sensing.

An additional aspect in the optimization of plasmonic biosensors is the low-concertation detection. In this regime, single binding events are notably rare. Therefore, it requires us to monitor many particles simultaneously for single-particle–single-molecule interactions to gather enough statistics for the reliable determination of concentration.^24^ Optimizing such a many-particle sensor consists of two main components: the optical characteristics of sensing elements and the transport of analyte from the bulk volume to the sensor surface. Here, we focus on the refractive index sensitivity, as the overall biosensing performance was found to be a product of two factors, optical performance and the rate of analyte transport.^25,26^

In this paper, we present a comprehensive computational study on the optimization of the size of gold nanorods for single-molecule plasmonic sensing in terms of optical refractive index sensitivity. We define contrast-to-noise ratio (CNR) as a universal measure of sensitivity, hence a universal figure of merit for plasmonic biosensing, which describes how effectively can a single molecular binding be resolved. We calculate the CNR for three distinct sensing schemas, namely, spectral sensing, fixed-wavelength scattering sensing, and fixed-wavelength absorption sensing. The goal of this paper, however, is not to discern between the three distinct detection techniques, as this would depend immensely on the experimental setup, but rather to provide the optimization route for each technique separately. Finally, we examine the impact of geometrical and material parameters used in our biosensor model on the sensing performance.

## 2 Methods

### 2.1 Biosensor Model

**Figure 1:**
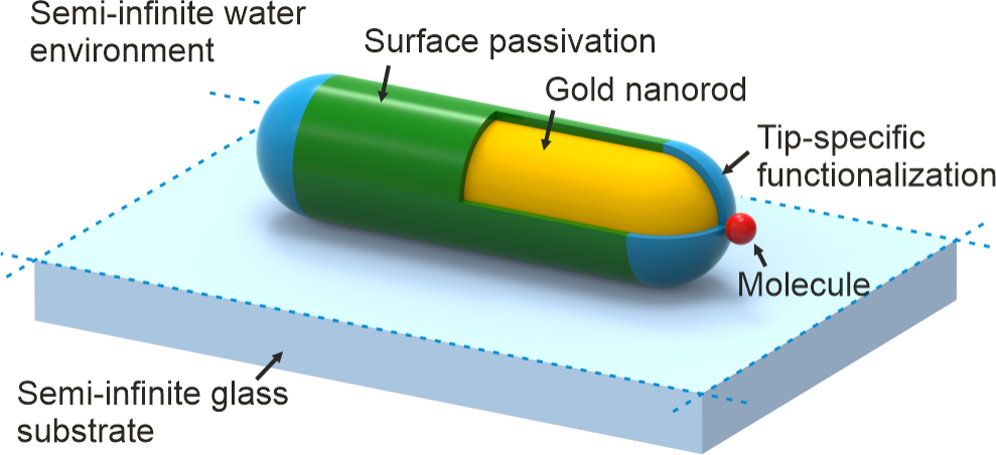
Geometry of a single-molecule plasmonic biosensor used in the computational model. A gold nanorod covered with a dielectric shell is placed on a semi-infinite glass substrate and immersed in a semi-infinite water environment. The shell resembles the tip-specific functionalization and passivation of the nanorod. The protein molecule is modeled as a dielectric sphere attached to the tip of the nanorod.

The geometry and material parameters used for our computational model of a single-molecule plasmonic biosensor are chosen to closely resemble the experimental conditions and commonly used biotin-streptavidin affinity system. The geometry is sketched in Figure 1. We simulate gold nanorods as spherically capped cylinders, which is a thermodynamically favored nanorod shape and can be synthesized with high monodispersity using current wet chemistry methods.^15,27^ The nanorod is covered with a thin dielectric shell and placed on a semi-infinite glass substrate, and immersed in a semi-infinite water environment. We use experimental values of the complex refractive index of gold by Johnson and Christy^28^ and constant refractive indices for all other materials. For the optimization of the size of nanorods, their width is varied from 10 to 40 nm and the aspect ratio (AR) from 2 to 5.

The shell mimics the functionalization and passivation layer of a tip-specific functionalization method, which is essential for high-sensitivity and high-specificity sensors.^17^ The method allows for selective functionalization of the tips and passivation of the “flat” part of the nanorod. This enables specific molecular binding only at sites, which provide high near-field enhancements. Here, we assume the shell thickness of 1.5 nm corresponding to the length of methyl-PEG4-thiol often used as a passivation agent. The same thickness can be assumed for the tips functionalized with biotin-PEG4-thiol as the biotin molecule is fully embedded in the binding pocket of the protein.^29^ We take the refractive index of the shell equal 1.45 as for pure PEG^30^ for both regions.

The analyte molecule is modeled as a dielectric sphere, which can be attached to the shell at virtually any position. In this study, we focus on small protein molecules; thus, we use streptavidin with a molecular weight of ∼52.8 kDa as our model molecule. Assuming a closely packed protein interior and protein density of 1.37 g/cm^3^,^31^ we model the streptavidin molecule as a sphere with a diameter of 5 nm. We use a refractive index of 1.57 as for the water-free streptavidin.^32^

For the optimization of the sensing performance, the molecule was always attached at the tip of the nanorod to overlap with the hotspot of electric field enhancement. As we aim for sensing in a low-concertation regime, for which binding events are notably rare, no more than one biding per nanoparticle is expected. As such, the measurement of concentration would be done by simultaneously monitoring many single-nanoparticle sensors allowing us to gather enough statistics in a reasonable time frame.^19^ Considering such a model, we optimize the sensitivity of single nanorods at their hotspot, as this allows for the most reliable detection of a single binding event. The total active area of a biosensor, and thus its dynamic range, can be varied by the number of particles that are being monitored, which is limited by the field of view and spatial resolution of a microscopic system.

### 2.2 Computational Details

To evaluate the sensing performance of single gold nanorods, we first calculate their plasmonic response upon binding an organic molecule (LSPR spectra with and without molecule), and second, we calculate the maximum measurable signal and the corresponding detection noise for each sensing modality to evaluate the contrast-to-noise ratio. The nanorods are illuminated from the top by a plane wave polarized along their long axis providing excitation of particle’s longitudinal LSPR mode.

The far-field scattering and absorption cross-sectional spectra (*C*_scat_ and *C*_abs_, respectively) are calculated using discrete dipole approximation (DDA) method, implemented in the ADDA software package,^33^ often used for numerical simulations of single plasmonic nanoparticles.^17,20,34,35^ In brief, DDA relies on discretizing a target particle into a set of small polarizable cubic subvolumes (voxels). Each voxel represents a point dipole that interacts with an external field and the other dipoles. The interactions form a set of linear equations, which can be solved iteratively for the polarization of each dipole. The optical properties of each voxel are defined by the complex refractive index allowing for any arbitrary shape and composition of the particle. The ADDA implementation also allows placing a particle near a semi-infinite dielectric surface, e.g., glass substrate.^36^ All calculations were run using GPU accelerated computing. The available GPU memory sets the limit to the total number of dipoles; thus, the size of dipoles was 0.25 nm for nanorods with a diameter 20 nm or smaller and 0.5 nm for bigger nanorods.

The accuracy of DDA depends on the ratio of voxel size to the wavelength and to the refractive index. Additional errors arise from the approximation of particle’s curved surfaces with cubic voxels and thus depend on the size of nanorods. As the computational errors are wavelength-dependent, we estimate the spectral position, maximum cross section, and resonance width of the LSPR by fitting the Lorentzian curve to the cross sections calculated for several wavelengths near the resonance peak. A detailed study of accuracy is presented in the Supporting Information.

### 2.3 Figures of Merit in Plasmonic Sensing

**Figure 2:**
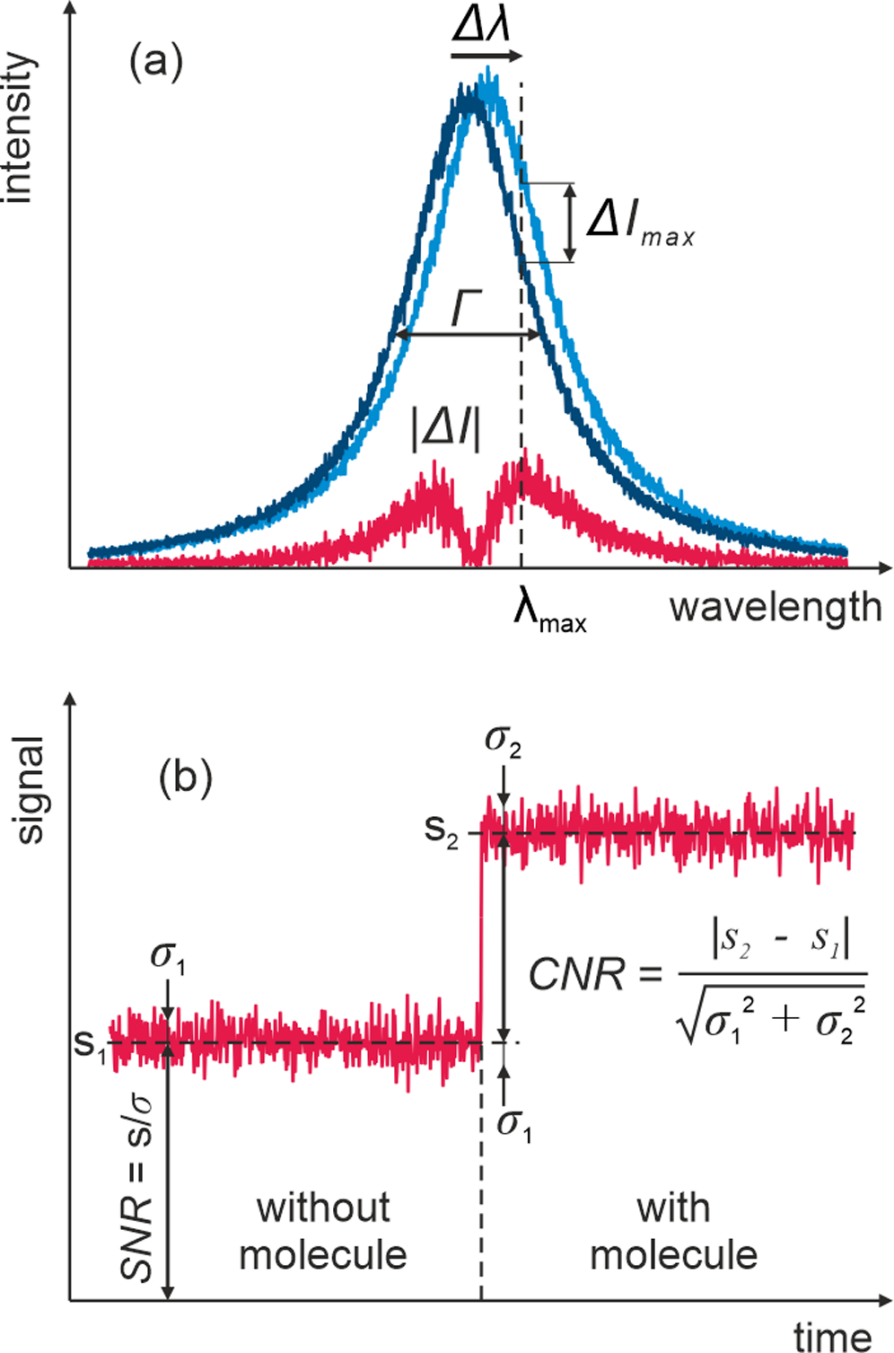
Illustration of measurable quantities in plasmonic biosensing. (a) Plasmon resonance of a nanoparticle is shifted upon binding an analyte molecule causing a local change of the refractive index. The shift can be detected directly by the spectroscopic measurement of Δλ, or indirectly by measuring the change of scattering or absorption intensity Δ*I* at a fixed wavelength. The maximum of Δ*I* is located at the long-wavelength side of the LSPR spectrum, close to the steepest point of the slope, i.e., λ_max_. (b) Either of the three quantities can serve as a measurable signal *s*, which is monitored over time for a single nanoparticle. The binding event is detected as a step function, provided that the step is higher than the respective measurement noise σ. The graph illustrates the difference between signal-to-noise ratio (SNR) and contrast-to-noise ratio (CNR). Γ, full width at half-maximum.

The shifts of plasmonic resonance induced by a molecular binding can be detected directly by the spectroscopic measurement or indirectly by measuring the change of scattering or absorption intensity at a fixed wavelength. These measurable quantities are sketched in Figure 2a. In addition to the resonance shift, the local change of refractive index near a plasmonic particle induces an increase of scattering and absorption cross sections and a broadening of the resonance. Thus, the wavelength at which the intensity change reaches maximum (λ_max_ and Δ*I*_max_, respectively) is located at the long-wavelength side of the LSPR spectrum, close to the steepest point of the slope.

**Figure 3:**
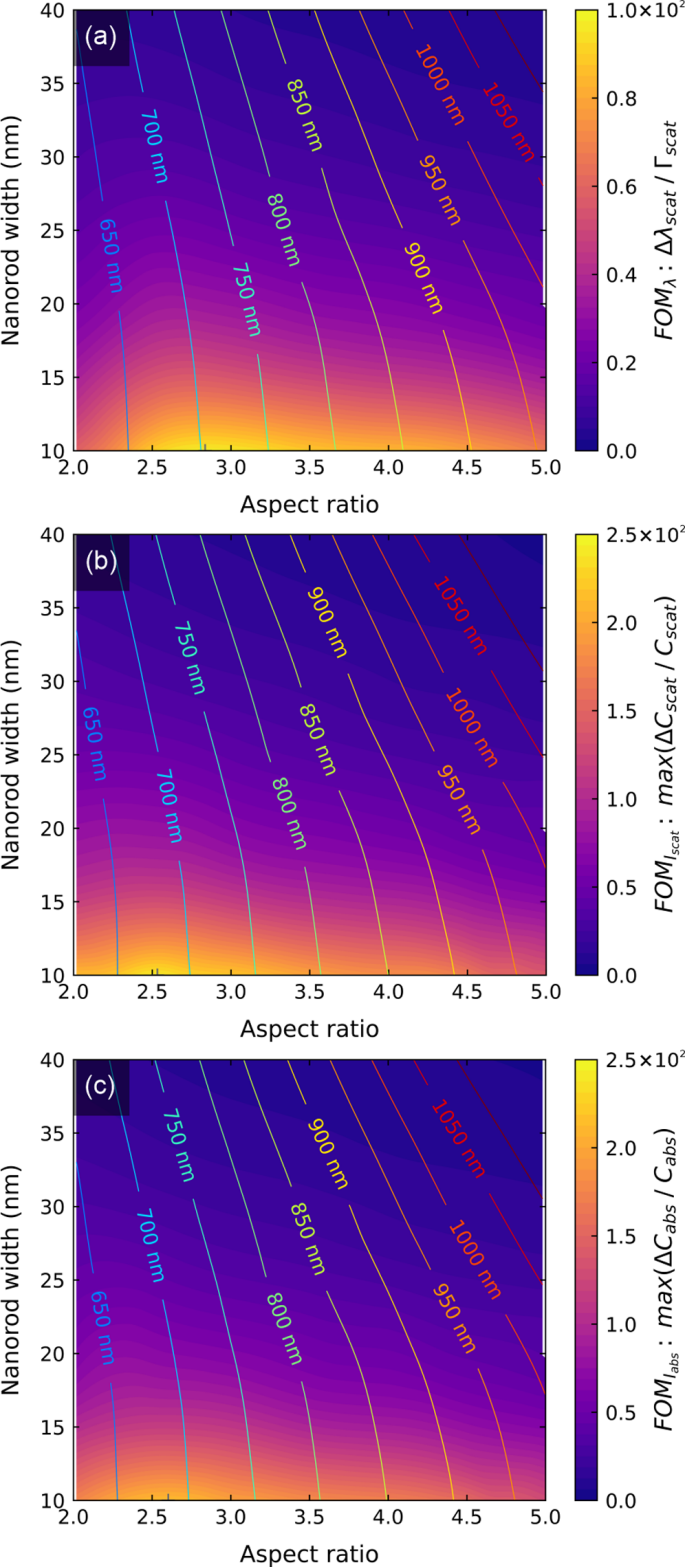
Noise-independent figures of merit (FOMs) for single-molecule plasmonic sensing in three modalities: (a) scattering-based spectral sensing, (b) scattering-based fixed-wavelength sensing, and (c) absorption-based fixed-wavelength sensing.

The standard noise-independent FOMs for plasmonic sensing are defined for spectral and fixed-wavelength modalities. The first is defined as a ratio of resonance shift per unit of bulk refractive index change to the spectral width of the resonance (Γ).^22^ For single-molecule sensing, the bulk sensitivity can be replaced by the maximum spectral shift induced by the binding of a single molecule; thus
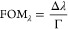
1The second standard FOM is defined for the fixed-wavelength sensing, simply as a maximum of a relative intensity change
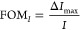
2The standard noise-independent FOMs calculated for single-molecule detection in scattering and absorption are plotted in Figure 3a–c. We found the highest values of all three FOMs for 10 nm wide GNRs, though their respective maxima appear at slightly different aspect ratios—at AR = 2.8 for FOM_λ_, AR = 2.5 for FOM_*I*scat_ and AR = 2.6 for FOM_*I*abs_. In all cases, a further increase of AR above the maximum causes a slight decrease of FOMs followed by a broad plateau. However, the maximum values drop significantly with increasing nanorod width.

The noise-independent FOMs would suggest that the optimum size for biosensing is approximately 10 × 25 nm^2^ for all methods. However, the optical response of such small plasmonic nanoparticles is dominated by absorption as the absorption is approximately proportional to particle’s volume and scattering to volume squared. This is a substantial obstacle for scattering-based detection, especially when other scattering species are present in the sample.^37,38^ Therefore, the ability to detect single organic molecules by plasmonic nanoparticles relies on two essential factors, first, high signal-to-noise detection of single particles, and second, precise measurement of the LSPR shift. As such, the measurement noise is critical for the sensing performance and should be accounted for in a properly defined FOM.

In the experiment, the plasmonic response of a single particle is monitored over time, and the discrete binding events are detected as a step function in the recorded signal, provided that the height of this step is larger than the respective measurement noise. Consequently, the sensitivity of single-molecule detection and a universal figure of merit can be defined as the contrast-to-noise ratio (CNR)
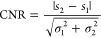
3where |*s*_2_ – *s*_1_| is the absolute difference of time-averaged signal with and without a molecule, respectively, while σ_1_ and σ_2_ are the respective root-mean-square noise.

The signal in eq 3 can be derived from the resonance shift or the change in scattering or absorption intensities. However, regardless of the detection technique used in the experiment, we can derive general equations for the registered signal and the related noise. The signal is proportional to the number of photons scattered directly by the nanoparticle (or indirectly by a thermal lens created by the photothermal effect), which are subsequently collected and transmitted by the optical setup, and finally registered by the detector within the integration time. Thus
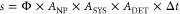
4where Φ is the illumination photon flux density (number of photons per second per area per wavelength unit, equal to *I*_0_/*hν*, with *I*_0_ being is the illumination intensity), *A*_NP_ is a wavelength-dependent scattering factor with unit of area, *A*_SYS_ is a dimensionless optical system factor (percentage of photons which are collected and transmitted through the system), *A*_DET_ is the wavelength-dependent external quantum efficiency of the detector, and Δ*t* is the integration time.

The size of nanorods influences the registered signal through the scattering factor, *A*_NP_, as both *A*_SYS_ and *A*_DET_ are essentially constant for a given experimental setup. Notably, for the background-free scattering-based detection, *A*_NP_ is equal to the scattering cross section. On the contrary, *A*_SYS_ depends mostly on the numerical aperture (NA) of the microscope objective, the reflections on optical elements, and the residual optical aberrations. For the sake of simplicity, we will assume a perfect optical setup (*A*_SYS_ = 1) and perfect detector (*A*_DET_ = 1), meaning that all scattered photons are collected and registered by the setup. As such, we neglect the impact of the angular distribution of far-field scattering, which in general is size-dependent, and thus for a given NA of the microscope objective can influence the signal. This assumption is justified because the detection of single nanoparticles requires high optical resolution, hence requires using high numerical aperture optics, and thus most of the forward or backward scattered light is collected by the objective regardless of the slight changes in its angular distribution.

The measurement noise has three main components, the optical system noise, σ_sys_, the detector noise, σ_det_, and the shot noise, σ_s_
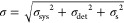
5σ_sys_ depends on the design of a particular optical setup and arises from the light sources (i.e., laser noise), vibrations, airflow, etc. The detector noise of a CCD or CMOS camera results from the temperature-dependent dark current, *I*_D_, and the read noise, σ_R_
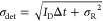
6The values of *I*_D_ and σ_R_ might vary significantly depending on the sensor and camera construction. The shot noise is associated with the randomness of photons arriving at the detector, governed by the Poisson statistics, which gives
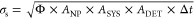
7Therefore, the size of nanorods has a strong influence on the measurement noise through the shot noise, particularly at signal levels allowing for a shot-noise-limited detection. With a scientific-grade CCD or sCMOS camera, the shot-noise-limited detection is typically reached for an input signal of about 100 photons per pixel within the integration time. In the optimization of the size of nanorods discussed in the following sections, we will assume a perfect optical setup (*A*_SYS_ = 1) working in the shot-noise-limited detection. As such, the signal-to-noise ratio (SNR) is given by
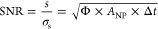
8The SNR values calculated for single nanorods with varying size are plotted in Figure S6 in the Supporting Information.

## 3 Results and Discussion

### 3.1 Optimization of the Size of Gold Nanorods under Constant Illumination Intensity Conditions

#### 3.1.1 Spectral Sensing

In the spectral sensing, the entire LSPR spectrum of a single nanoparticle is measured continuously, and the discrete molecular binding events are detected as step-like resonance shifts. Experimentally, it is facilitated by dark-field microscopy and an intense broad-band illumination (e.g., supercontinuum white light laser). The light scattered by a single particle is directed to a spectrometer equipped with a line CCD sensor providing high temporal resolution.^18^

**Figure 4:**
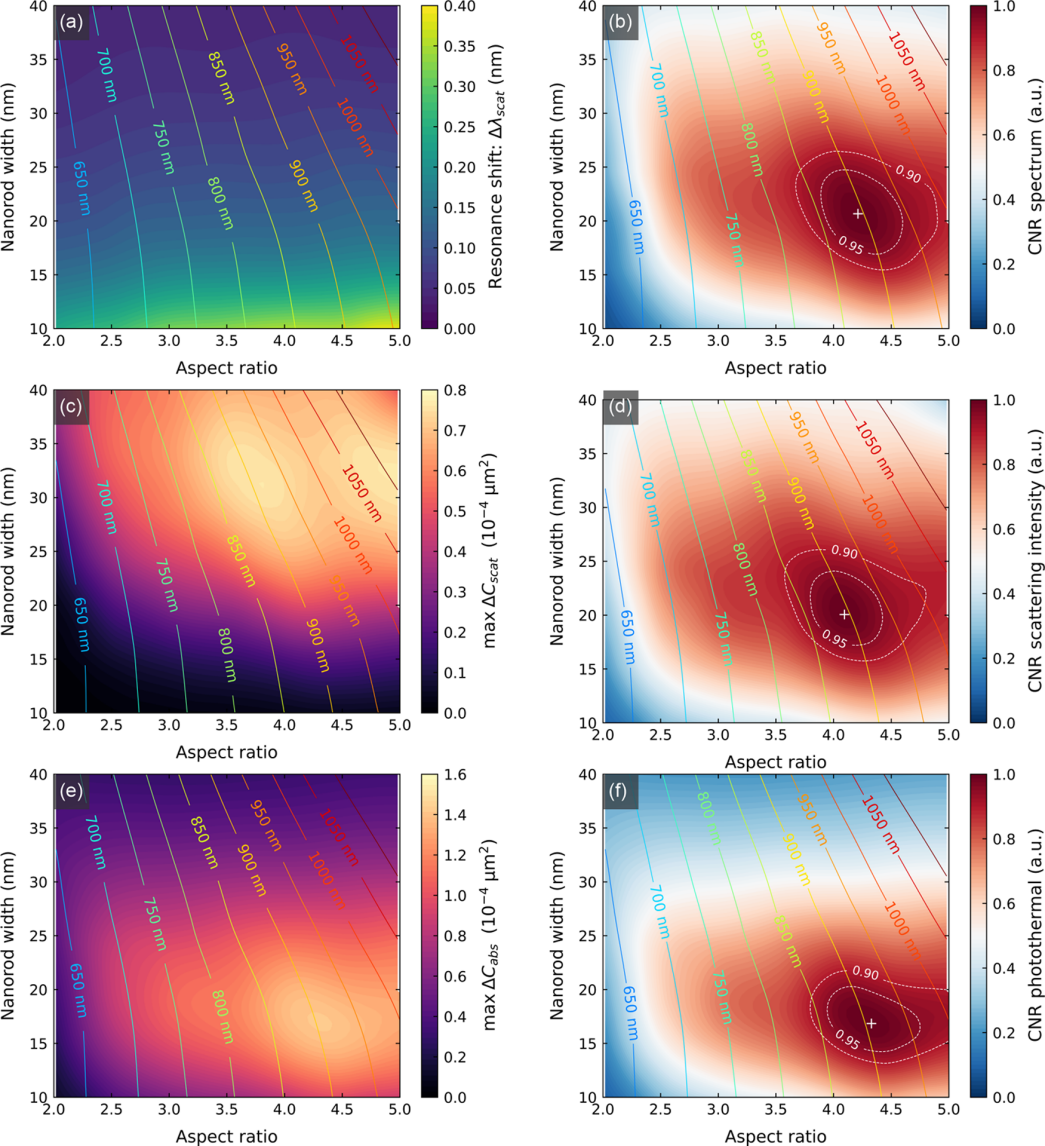
Optimization of the size of gold nanorods for single-molecule plasmonic sensing under constant illumination intensity conditions. The left column depicts the signal, and the right column corresponds to contrast-to-noise (CNR) ratios for (a, b) spectral, (c, d) fixed-wavelength intensity, and (e, f) fixed-wavelength photothermal sensing. The solid-line contours show the wavelength at which respective quantities have maximum vales for a given nanorod size. The white dashed lines mark the area in which CNR holds values above 90 and 95% of the maximum marked with “+” sign.

The registered signal is given directly by the resonance position, which is determined from the fitting of the Lorentzian peak to the measured spectrum. Thus
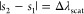
9Figure 4a depicts the dependence of the resonance shifts on the size of GNRs. We found the Δλ_scat_ to increase steadily with increasing aspect ratio and decrease rapidly with increasing width of the nanorods; thus, the maximum shift is provided by the nanorods of 10 × 50 nm^2^. Notably, its maximum value is only 0.39 nm for a simulated ∼53 kDa protein.

The measurement noise at each wavelength results in the uncertainty of peak fitting. Following the derivation by Nusz et al.,^23^ the fitting noise, σ_fit_, is directly proportional to the resonance width (Γ) and inversely proportional to the SNR of the spectral measurements, with a proportionality factor of η = 0.21
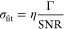
10For a shot noise-limited measurement, we obtain
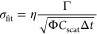
11Based on eq 3, the CNR_λ_ for spectral sensing can be calculated as
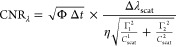
12Interestingly, when assumed that the increase of Γ and *C*_scat_ associated with the resonance shift is insignificant compared to the impact of Δλ_scat_, eq 12 simplifies to
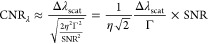
13Consequently, up to a multiplicative constant, CNR_λ_ is proportional to the standard noise-independent FOM_λ_ for spectral sensing multiplied by the SNR of single-particle detection
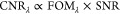
14Figure 4b depicts the dependence of CNR_λ_ on the size of GNRs calculated (according to eq 12) for constant illumination intensity, i.e., the same value of intensity for all sizes. The values of CNR are normalized to the maximum to illustrate the percentage drop of sensing performance with respect to the optimum. We found a clear maximum in CNR_λ_ located at the size of 21 × 87 nm^2^ corresponding to the resonance wavelength of approximately 900 nm. The existence of the maximum can be understood as a competition between two contradictory effects. First, the resonance shift increases with decreasing width and increasing aspect ratio; and second, the SNR of single-particle detection increases with increasing GNR volume. As such, smaller GNRs provide narrower resonance and larger resonance shifts but scatter less light, so they are more difficult to detect.

#### 3.1.2 Fixed-Wavelength Scattering Intensity Sensing

In fixed-wavelength sensing, the discrete molecular binding events can be detected as step-like changes of the registered scattering intensity. Experimentally, the detection is facilitated by the background-free scattering microscopy, i.e., dark-field or total internal reflection configurations. The light scattered by single nanoparticles is simply registered by a two-dimensional (2D) microscope camera with a frame rate sufficient to resolve molecular binding in time. This technique allows for monitoring large numbers of single particles simultaneously substantially improving the dynamic range of the biosensor.^19^

According to eq 4, the difference in registered intensity at sensing wavelength is governed the change of scattering cross sections
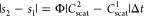
15The dependence of maximum change of scattering cross section on the size of GNRs is depicted in Figure 4c. We found a broad maximum between the widths of 25–40 nm and aspect ratios between 3 and 5, with two local peaks located at the sizes of 32 × 119 and 32 × 160 nm^2^. The maximum value is 0.76 × 10^–4^ μm^2^.

For an ideal experimental setup, the total measurement noise is given by the shot noise
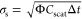
16Consequently, based on eq 3, the CNR for fixed-wavelength sensing can be calculated as
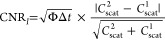
17Interestingly, when assumed that the increase of Γ and *C*_scat_ associated with the resonance shift is insignificant compared to the impact of Δ*C*_scat_ on the CNR, eq 17 simplifies to
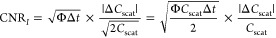
18Up to a multiplicative constant, CNR*_I_* is proportional to the standard noise-independent FOM*_I_* for fixed-wavelength sensing multiplied by the SNR of single-particle detection
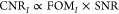
19The calculated dependence of CNR*_I_* (according to eq 17) on the size of GNRs is depicted in Figure 4d. We found a clear maximum located at the size of 20 × 82 nm^2^. The maximum value of CNR was found for the sensing wavelength of approximately 890 nm, located at the long-wavelength side of the nanorod’s LSPR spectrum. As before, this maximum results from a trade-off between two competing effects. First, the sensing performance depends on the effectiveness with which the resonance shift can be detected. In the case of fixed-wavelength sensing, this is governed by the FOM_I_ reaching a maximum value at 10 × 25 nm^2^. Second, the sensing performance depends on the SNR for single-particle detection, which increases with nanoparticle volume.

#### 3.1.3 Fixed-Wavelength Photothermal Sensing

Single-molecule plasmonic sensing based on the photothermal effect exploits the changes of power absorbed by a nanoparticle resulting from the shifts of plasmonic resonance. In the experiment, two laser beams of different wavelengths are collinearly focused on the sample. The intensity-modulated heating (pump) beam at λ_max_ is absorbed by the nanoparticle and released in the form of heat. In turn, the local change of temperature induces periodic changes of refractive index in the surrounding medium. Such time-dependent thermal lens influences the second (probe) beam at wavelength outside of the LSPR spectrum. The observable signal arises from the interference between the field scattered by the thermal lens and the unperturbed reference field. It is commonly detected using lock-in amplification.^20,39^

The detailed description of nanoscale heating by time-variable beam and the derivation of photothermal signal magnitude is a complex problem. It depends on the configuration of the experimental system (transmission vs. reflection, scanning vs. wide field, etc.), the collective temperature response of a nanoparticle assembly, modulation frequencies, spatial modes of scattered and reference fields, heat transfer dynamics, and thermal properties of the surrounding medium.^39,40^ Thus, for the sake of simplicity, we introduce a photothermal scattering factor, *C*_th_, which describes the scattering cross section of the time-varied thermal lens per watt of dissipated power (assuming that the properties of the thermal lens are dominated by the absorbed power)
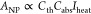
20where *C*_abs_ is the absorption cross section and *I*_heat_ is the illumination intensity of the heating beam. Accordingly, the change in observable signal is equal to
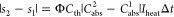
21and is governed by the change in absorption cross section at heating beam wavelength. The maximum change of *C*_abs_ is plotted in Figure 4e. We found a single-peak maximum, elongated in the aspect ratio axis, located at the nanorod size of 17 × 73 nm^2^. The maximum CNR was found for the sensing wavelength of approximately 900 nm (pump beam), located at the long-wavelength side of the nanorod’s LSPR spectrum.

As the photothermal signal is derived from the interference between strong reference field and weak scattering field the noise of photothermal signal is independent of the heating beam intensity and depend only one the probe beam (illumination photon flux density) and the integration time
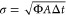
22with *A* being the area of the detector. Based on eq 3, the CNR_P_ for fixed-wavelength photothermal is linearly dependent on the maximum change of *C*_abs_
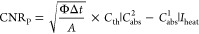
23The calculated dependence of CNR_P_ on the size of GNRs is depicted in Figure 4f. As the noise in photothermal sensing is independent of the heating beam, CNR_P_ reaches the maximum at the same size as Δ*C*_abs_, i.e., 17 × 73 nm^2^.

#### 3.1.4 Discussion

Let us now discuss obtained optimization results. First, a clear maximum of sensing performance exists for each sensing modality. The observed maxima are relatively broad. As a result, the size parameters space (area) for which the CNR reaches values above 95% of the maximum (marked with dashed white lines in Figure 4) coincides with an 8% size deviation in both width and length. Notably, this level of size monodispersity is currently achieved with wet chemistry synthesis of GNRs.^15^

It is important to note that as we assume an ideal experimental setup and an ideal detector, the observed maxima of sensitivity originate inherently form the plasmonic properties of gold nanorods. Therefore, with the goal to detect the smallest molecules (i.e., below 50 kDa) that produce tiny resonance shifts easily obscured by noise, the experimental setup should be optimized to reach shot-noise-limited performance in the spectral range matching the response of optimized nanorods.

A fundamental characteristic for a nonideal experimental system is the quantum efficiency (QE) spectrum of the detector as the CNR is proportional to . This relation is apparent from eqs 4 and 7. Therefore, the QE should be spectrally matched to the sensing wavelength, which according to calculations is approximately 900 nm. At this spectral range, deep-depleted CCD sensors, which are optimized for near-infrared range, would provide a good match. The use of standard silicon CCD or CMOS sensors optimized for visible light would favor the nanorods with lower aspect ratios which have a resonance closer to the maximum QE of the detector. The values of CNR corrected for detectors’ QE are plotted in Figures S8 and S9 in the Supporting Information. Importantly, this problem does not affect the photothermal detection technique, as in this case, the wavelength of pump laser beam can be matched with the plasmonic resonance independently of the probe beam, which can be matched with the maximum QE of the detector.

### 3.2 Impact of Model Parameters on the Sensing Performance

The optimization of the size of nanorods presented in the previous sections was done for the standard parameters of our biosensor model described in detail in Section 2.2. The CNRs, however, are expected to depend strongly on the analyte molecule, i.e., its size (molecular weight), shape, refractive index, and binding position, as well as on the functionalization and passivation of the nanorod, i.e., thickness and refractive index of the shell. We will now examine the impact of each parameter one by one while keeping the remaining parameter values as in the original model. We choose a nanorod size of 20 × 80 nm^2^ as close to the optimum size for all three detection schemas.

**Figure 5:**
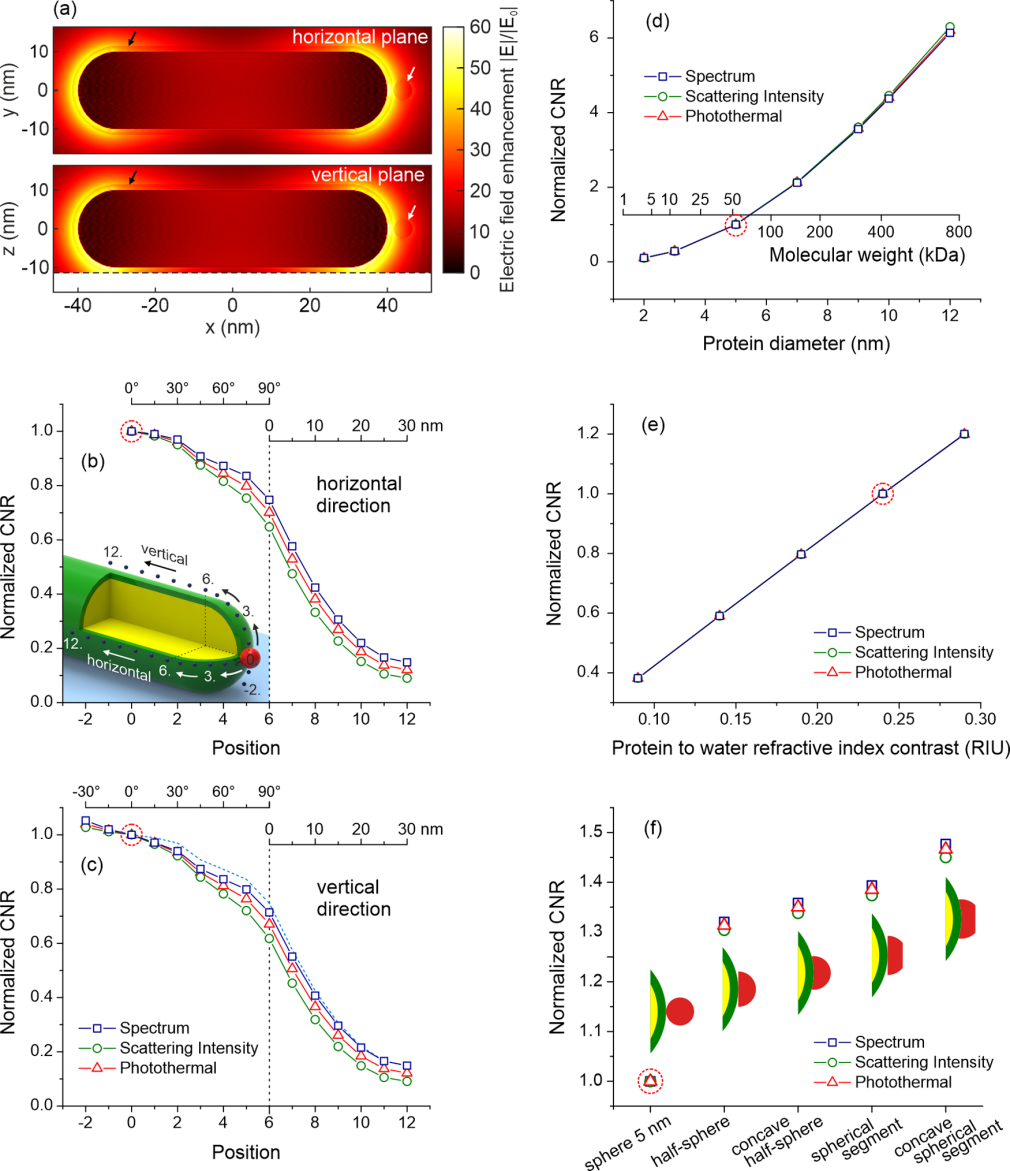
Impact of parameters used in the numerical model on the biosensing performance. (a) Electric field enhancement near a gold nanorod 20 × 80 nm^2^ with a 1.5 nm thick functionalization layer on glass and dielectric nanosphere attached at the tip in a horizontal (parallel to the substrate) and vertical (perpendicular to the substrate) planes. White arrows point to the molecule, while black arrows indicate the nanorods’ shell. (b, c) Impact of a protein binding position on the contrast-to-noise ratio (CNR) normalized to the case of protein attached at the tip of the nanorod. Binding sites are uniformly distributed along (b) the side (horizontal plane) and (c) the ridge (vertical plane) of the nanorod as illustrated in the inset sketch. Position “0” marks the tip, and position 12 marks the middle of the nanorod. The blue dashed line in (b) represents values of CNR for spectral sensing in horizontal positions for direct comparison. (d–f) Impact of protein volume (d), protein refractive index (e), and protein shape (f) on the contrast-to-noise ratio (CNR) for spectral, fixed-wavelength, and photothermal sensing. The CNRs are normalized to the parameters used previously for the size optimization (marked with red circles). The molecular weight in (d) assumes closely packed protein interior and protein density of 1.37 g/cm^3^. The diameters and heights for each shape presented in (f) are adjusted to have the same volume as 5 nm diameter sphere.

In general, the resonance shift depends on the overlap integral between the analyte molecule and the local field enhancement.^6^ The local electric field enhancement near a nanorod of 20 × 80 nm^2^ with a molecule attached at its tip is plotted in Figure 5a. As expected, the field enhancement in a horizontal plane (parallel to the substrate) is axisymmetric with two hotspots located near the ends of the nanorod. However, the presence of glass substrate breaks the rotational symmetry, and consequently, in the vertical plane, the field enhancement is asymmetric and increases toward the substrate. Additionally, the field decays rapidly with the distance from the nanorod, hence, the region with the highest enhancement is mostly confined within the shell.

The inhomogeneity of field enhancement around a nanorod results in a strong dependence of the sensing performance on the analyte binding position. Figure 5b,c depicts the impact of binding position on the CNR for spectral, fixed-wavelength, and photothermal sensing normalized to position 0 (tip of the nanorod). Binding sites are uniformly distributed every 15° on the curved part of the nanorod and every 5 nm on the flat part so that position “12” marks the middle point of the nanorod. Due to the asymmetry of field enhancement, we investigated binding sites along the side and the ridge of the nanorod separately, as illustrated in the inset sketch of Figure 5b. For all sensing techniques, we observed a steady decrease of CNR with the molecule moving away from the tip on the spherical part of the nanorod, followed by a rapid drop of CNR with the molecule moving further away from the ending on the nanorod’s flat side. Interestingly, spectral sensing was found to be the least affected by the binding position. In the middle of the nanorod (position 12), the calculated values of CNR are from 85 to 91% lower than at the tip, for spectral and fixed-wavelength scattering sensing, respectively. This effect is expected to be less pronounced for shorter nanorods as the hotspots are closer together providing higher field enhancement in the middle.

The rapid drop of CNR between positions 6 and 12 reveals that the passivation of the flat part of the nanorod, hence the low-sensitivity areas, is the key to achieve high-sensitivity and high-specificity sensors. In general, short nanorods provide high ratio of high- to low-sensitivity area, which corresponds to the area of the tip and the sides of the nanorod, respectively. On the other hand, longer nanorods provide higher field enhancement at their tips, however accompanied by a substantial increase of the low-sensitivity area. This trade-off can be resolved by the passivation. A superior signal quality is achieved by exploiting the high field enhancement provided by long nanorods without compromising the overall biosensing performance by the increased probability of molecular binding in the large-area low-sensitivity regions on the sides^17,41^

The interaction between plasmon and the substrate results in slightly higher values of CNR for the horizontal direction compared with the vertical direction at corresponding positions. The dashed line in Figure 5c shows values of CNR for spectral sensing in horizontal positions for direct comparison. The biggest differences are observed at positions “4” and “5” reaching 3.7% for the spectral sensing technique. In the vertical direction, binding sites close to the glass provide higher values of CNR than at the tip. The maximum improvement of 5% was observed for the spectral sensing schema in position “–2.” However, binding at this site is not likely because of the low accessibility of fluid constricting the diffusion of analyte molecules.

The next three parameters to impact the sensing performance are the size, the refractive index, and the shape of the molecule. The calculated dependencies of CNRs for the three sensing modalities are depicted in Figure 5d–f. Notably, all sensing modalities show almost identical dependence on the three parameters.

Figure 5d depicts the impact of molecule diameter (assuming spherical shape) on the CNR normalized to the diameter of 5 nm used for the optimization of size of nanorods. We found a substantial increase of CNR in the diameter range between 2 and 12 nm corresponding to the molecular weight ranging from 3.6 to 746 kDa (assuming density of 1.37 g/cm^3^). This dependence can be again explained by increasing overlap integral between the field enhancement and the molecule. However, as the field enhancement is rapidly decaying with the distance from the particle, the overlap integral does not scale linearly with the molecule volume, hence molecular weight. Instead, we found a power dependence of CNR on diameter with exponent equal to 2.3.

Figure 5e depicts the impact of the RI contrast (with respect to the water environment) on the CNR normalized to the RI contrast of 0.24. This dependence was found to be strictly linear.

In general, the shape of proteins is immensely complicated, and furthermore, it might not be preserved after binding to the nanorod. Thus, we investigated the impact of molecule shape on the biosensing performance by deviating the original assumption of its spherical shape toward shapes that are more closely glued to the surface of a nanorod. The volume of the molecule was kept constant while the shape changed. The calculated values of CNRs are depicted in Figure 5f together with the sketches of investigated shapes. We found up to a 47% increase of CNR when the shape of the molecule was changed from sphere to concave spherical segment. This effect also results from the increased overlap integral. The shape effects are expected to be more pronounced for larger molecules.

**Figure 6:**
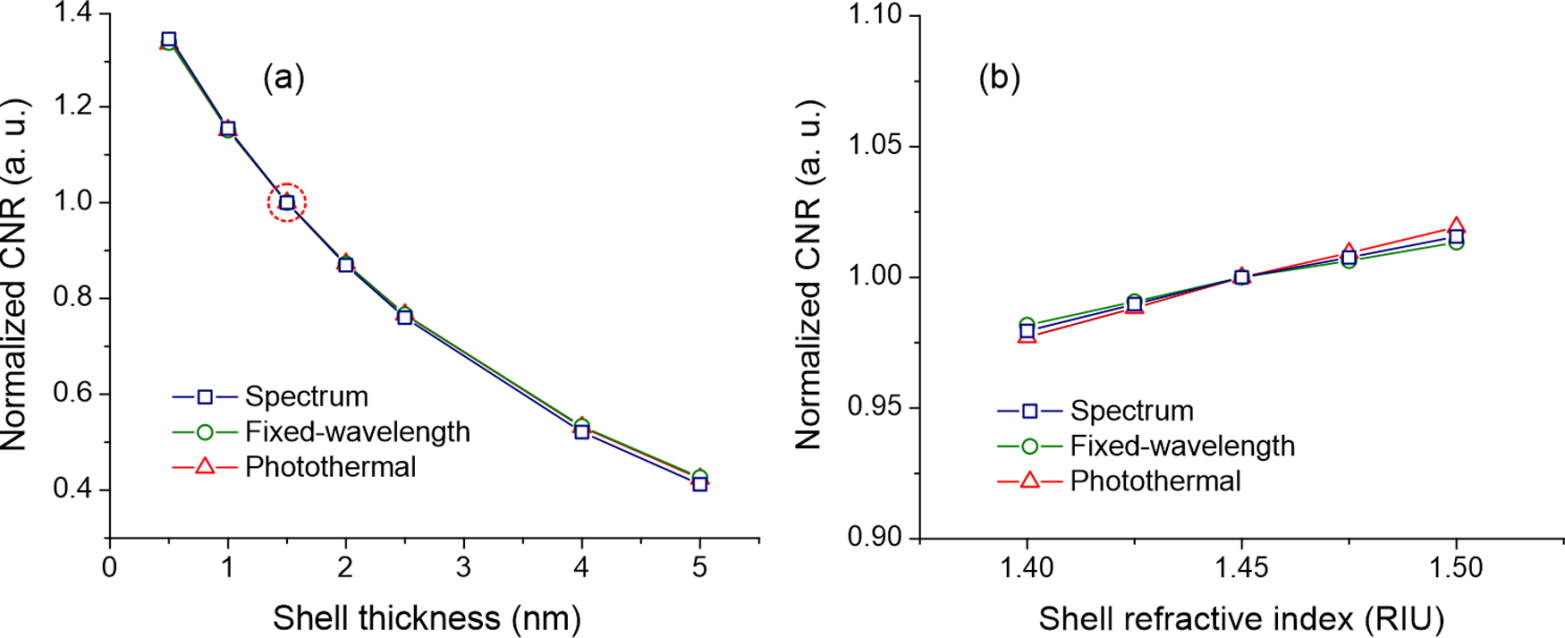
Impact of (a) shell thickness and (b) shell refractive index on the contrast-to-noise ratio (CNR) for spectral, fixed-wavelength, and photothermal sensing. The CNRs are normalized to the parameters used previously for the size optimization, marked with a red circle.

Finally, we investigated the impact of shell parameters on the sensing performance. Figure 6a depicts the impact of shell thickness, and Figure 6b depicts the impact of shell refractive index on the CNR for spectral, fixed-wavelength, and photothermal sensing. The CNR values are normalized to the thickness of 1.5 nm and RI of 1.45, respectively. First, we found an exponential decrease of CNRs with increasing shell thickness. As the shell becomes thicker, the molecule is attached further away from the nanorod resulting in a substantial decrease of the overlap integral. Second, the refractive index of the shell was found to have only minor effect on the sensing performance. The total difference of 4% was found with RI varied in a relatively wide range between 1.4 and 1.5.

Another factor which has an impact on CNR is the shape of nanorod. In our simulations, we assume a spherically capped cylinder as a thermodynamically favored shape. However, any deviations from this model shape are expected to influence the resonance spectral position and the distribution of near-field enhancement, which, in consequence, would influence the values of CNR. The broad subject of nanoparticle shape is beyond the scope of this work, though detailed studies can be found in the literature.^41−43^

### 3.3 Adjustment of Illumination Intensity According to the Steady-State Temperature Increase

The results presented in the previous sections, in particular the optimization of nanorod size, were obtained for constant illumination intensity. However, the absolute value of SNR is highly dependent on the scattered photon flux density. In principle, one could therefore adjust the illumination intensity to obtain desired SNR in a shot-noise-limited experimental setup. For instance, let us consider an optimum nanorod size for spectral sensing calculated previously for constant illumination (21 × 87 nm^2^). One should expect that smaller nanorods (lower diameter) would scatter less light, resulting in lower SNR, but provide larger resonance shifts. Consequently, if the illumination intensity can be increased to compensate for lower scattering, the absolute value of CNR can be increased influencing the optimum size of GNRs. In particular, if the intensity could be increased indefinitely, the optimum size would be independent of the SNR of nanoparticles detection, and therefore will be governed by the standard noise-independent FOMs. As such, we need to define a limiting factor for the maximum attainable power density. Most commonly, this limit is set by an acceptable increase of temperature around the nanoparticles, which does not lead to the degradation of analyte molecules.

The increase of temperature for a nanoparticle under illumination depends on the power absorbed by the nanoparticle, its geometry, and the thermal properties of the surrounding medium. The steady-state temperature increase of an arbitrary-shaped metallic particle at its surface can be calculated as^44^
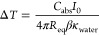
24where *R*_eq_ is the volume equivalent radius of a sphere (for spherically capped nanorods , where *D* is the length and *d* is the diameter), β is a universal dimensionless thermal capacitance coefficient equal to 1 for a sphere (tabulated values for nanorods calculated with boundary element method taken from Baffou et al.^44^), and *κ*_water_ is the thermal conductivity of the medium, i.e., water.

Several factors contribute to the overall temperature increase for a GNR of a certain size: first, the absorption cross section, hence the absorbed power scales approximately with nanoparticle volume. On the other hand, bigger particles are more effective in heat dissipation; thus, for a fixed absorbed power, their temperature is lower. Furthermore, β increases as the shape of a nanoparticle deviates from a spherical one. As a sphere is the least effective shape for heat dissipation, the increased surface-to-volume ratio, with respect to a sphere, leads to more efficient heat release from the particle and lower surface temperature.

**Figure 7:**
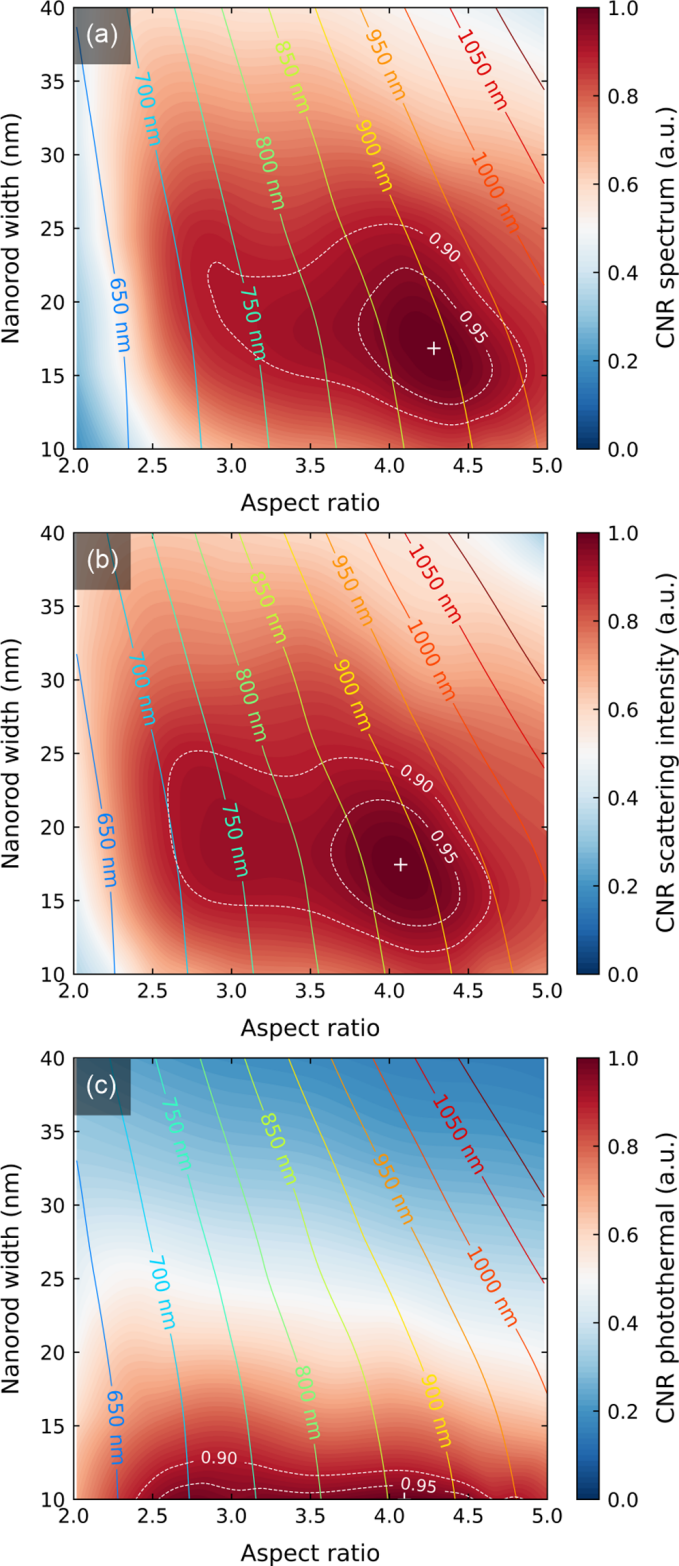
Impact of the size of nanorods on the normalized contrast-to-noise ratio for (a) spectral, (b) fixed-wavelength, and (c) photothermal sensing for illumination intensity normalized for constant steady-state temperature increase. Solid-line contours show the wavelength at which respective quantities have maximum values for a given nanorod size. The white dashed lines mark the area in which CNR holds values above 90 and 95% of the maximum marked with “+” sign.

Based on the above considerations, we calculate the impact of the size of GNRs on the CNR adjusting illumination intensity of each size to provide the same steady-state temperature increase. The results for spectral, fixed-wavelength scattering, and photothermal sensing are depicted in Figure 7a–c, respectively. The optimum sizes are summarized in Table 1. As for the constant illumination case, we found a maximum in the sensing performance of each technique. However, compared to the constant illumination, the optimum size of nanorods is shifted toward smaller nanorods.

**Table 1:** Optimum Sizes of Gold Nanorods for Constant Illumination Intensity and Constant Steady-State Temperature Increase for Spectral, Fixed-Wavelength, and Photothermal Single-Molecule Plasmonic Sensing

	constant illumination intensity (nm^2^)	constant temperature increase (nm^2^)
spectrum	21 × 87	17 × 72
fixed-wavelength	20 × 82	17 × 71
photothermal	17 × 73	10 × 41

The optimum size for scattering-based detection in the temperature-limited case can be understood considering that the absorption cross section of plasmonic nanoparticles is approximately proportional to the nanoparticle’s volume while scattering is proportional to the volume squared. As such, the response of small nanorods is dominated by the absorption, while for bigger particles, scattering is the dominant factor. The optimum size for scattering-based detection is therefore driven by a trade-off between the power scattered by the nanoparticle and power dissipated in the form of heat into the environment. This trade-off, however, does not affect the photothermal sensing. As such, in the temperature-limited case, the optimum size of GNRs changes substantially. CNR_P_ shows a broad plateau for AR ranging from 2.5 to 4.5 with a local maximum at 10 × 41 nm^2^.

Let us now discuss the implications of these results. Under low-illumination-intensity conditions, for which a temperature increase and resulting degradation of sample is not a concern, the optimum size of GNRs is determined by the constant power calculations (Figure 4b,d,f). If higher intensities are required (e.g., to provide a certain level of CNR), large GNRs with high absorption cross sections will start to heat up beyond the acceptable level. Once this condition is reached for the constant-intensity optimum size, the further increase of illumination intensity will result in a shift of optimum size toward smaller GNRs. At the illumination intensity for which all considered sizes are vulnerable to overheating, the optimum sizes are given by the variable illumination calculations (Figure 7a–c). As such, the optimum for moderate intensities lies between the two limiting cases. This effect is particularly pronounced for photothermal detection where there is no trade-off between absorption and scattering. Notably, if the illumination intensity could be increased indefinitely, the optimum size of nanorods becomes independent of the noise, and as such, it would be governed by the standard FOMs (Figure 3a–c).

## 4 Concluding Remarks

In this paper, we present a comprehensive computational study on the optimization of the size of gold nanorods for single-molecule plasmonic sensing in terms of optical refractive index sensitivity. We create an experimentally relevant model of a single-particle biosensor consisting of a spherically capped gold nanorod placed at the water/glass interface with tip-specific functionalization and passivation layers and biotin-streptavidin affinity system. We consider three distinct detection techniques, namely, spectral sensing, fixed-wavelength scattering sensing, and fixed-wavelength photothermal sensing. The ability to detect single organic molecules by plasmonic nanoparticles relies on two essential factors, first, high signal-to-noise detection of single particles, and second, precise measurement of the LSPR shift. Thus, we introduced a universal figure of merit, termed contrast-to-noise ratio (CNR), which relates the change of observable signal caused by discrete molecule binding events to the detection noise. As such, the CNR describes how well a single binding event can be resolved. By considering an ideal shot-noise-limited performance of experimental setups, we demonstrate the existence of maximum CNR for each sensing technique, resulting purely from the plasmonic properties of the nanorods. The optimization of nanorod size at constant illumination intensity yields surprisingly similar values for the three considered sensing techniques of about 20 × 80 nm^2^. For this size of a nanorod, we considered the impact of the geometrical and material parameters describing the molecule and the functionalization/passivation layer on the sensing performance. We found a substantial decrease of CNR with the molecular binding position moving away from the tip of the nanorod, a power dependence of CNR on molecule diameter with exponent equal to 2.3, the linear dependence of CNR on molecule refractive index. Additionally, we found an exponential decrease of CNR with increasing thickness of a functionalization/passivation layer (shell thickness) and insignificant dependence of CNR on its refractive index. All of these effects can be explained by considering changes in the overlap integral between the molecule and the electric field enhancement produced by the plasmon resonance. Finally, we discussed the thermal effects and the impact of nanoparticle heating on the optimum size of the nanorods described in our model. We describe two boundary cases. First, in low-illumination-power regime, the optimum size is defined by the constant illumination optimization. In the high-illumination-power regime, the optimum size is governed by the maximum temperature increase of the nanorod. This results in a minor shift of the optimum sizes toward smaller nanorods for the scattering-based sensing originating from the substantial dependence of the scattering and absorption cross sections on the size of nanorods. For photothermal sensing, the shift of optimum sizes is substantial as it does not depend on the trade-off between the absorption and scattering.

It should be indicated that our computational optimization is pertinent to the biosensor model used herein and focuses on the optical sensitivity for small refractive index change in the form of molecules. The complete optimization of a plasmonic biosensor is an inherently complex topic that would require a detailed description of several aspects, including (i) the mass transport between the bulk volume to the sensor surface, (ii) the exact spatial distribution of high-sensitivity binding sites on nanoparticle’s surface, (iii) the diffusion of analyte molecules on the nanoparticles surface, (iv) the thermal effects, e.g., thermophoretic forces, (v) various shapes of the sensing particles, (vi) the nanoparticles’ surface chemistry and its impact the plasmonic resonance, (vii) specific properties of analyte and receptors, and (viii) spatial distribution of nanoparticles for many-particle sensors. Nonetheless, the results obtained here show that the contrast-to-noise ratio is a valid universal measure of sensitivity and that the size of gold nanorods can be optimized purely due to the plasmonic properties of gold nanorods.

## Supporting Information Available

Accuracy of numerical methods; spectral position; maximum cross section and spectral width of localized surface plasmon resonance for scattering and absorption; signal-to-noise ratio as a function of nanorod size; and contrast-to-noise ratio corrected for the quantum efficiency of detectors (PDF)
