# Supplementary material for: Computational Optimization of the Size of Gold Nanorods for Single-Molecule Plasmonic Biosensors Operating in Scattering and Absorption Modes
Source: J Phys Chem C Nanomater Interfaces. 2021 Jul 1;125(27):14765–77. doi: 10.1021/acs.jpcc.1c02510 (PMC8411831; doi:10.1021/acs.jpcc.1c02510)
Supplement: Supplementary file 1 — jp1c02510_si_001.pdf [file jp1c02510_si_001.pdf]

Supporting Information
Computational Optimization of the Size of Gold Nanorods for Single-Molecule Plasmonic Biosensors Operating in Scattering and Absorption Modes

Teresa Staniszewska, Maciej Szkulmowski, and Seweryn Morawiec*

Institute of Physics, Faculty of Physics, Astronomy and Informatics, Nicolaus Copernicus University in Torun, Grudziadzka 5, 87-100 Torun, Poland
*seweryn.morawiec@fizyka.umk.pl, +48 56 611 3214

1 Study of accuracy

Systematic study of DDA (discrete dipole approximation) accuracy in case of gold nanoparticles in vacuum was presented by Yurkin et al.[1] Here we present our view on gold nanoparticles in water environment that involves a systematic error of the resonance position and an uncertainty of Lorentz profile fitting.

1.1 Systematic error

We compared extinction efficiency spectra (Q_{ext}) simulated by ADDA with Mie's solutions for gold nanospheres with diameters ranging from 10 to 40 nm. In figure S1. it is seen that simulated spectra qualitatively model shape of exact solution.

[1] Maxim A. Yurkin, David de Kanter, Alfons G. Hoekstra, *Accuracy of the discrete dipole approximation for simulation of optical properties of gold nanoparticles*, Journal of Nanophotonics **4** (2010)

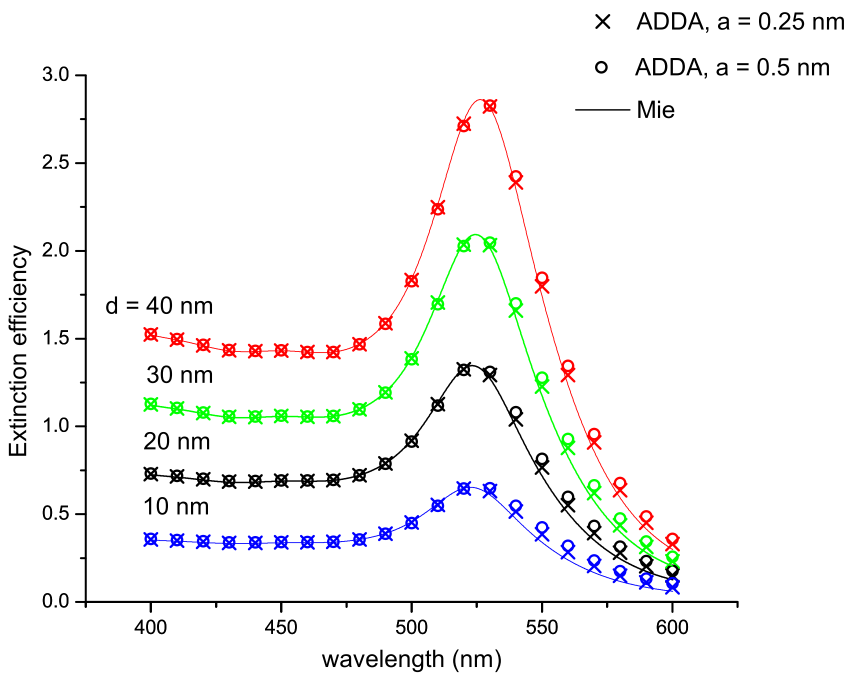

Figure S1: Comparison of extinction efficiency obtained from ADDA and Mie's solution for four spheres with diameter from 10 to 40 nm. For calculations in ADDA two dipole sizes were applied - 0.25 and 0.5 nm.

Detailed comparison of these spectra are presented in figure S2. that shows differences of efficiencies calculated in both methods (i.e. $Q_{ADDA} - Q_{Mie}$).

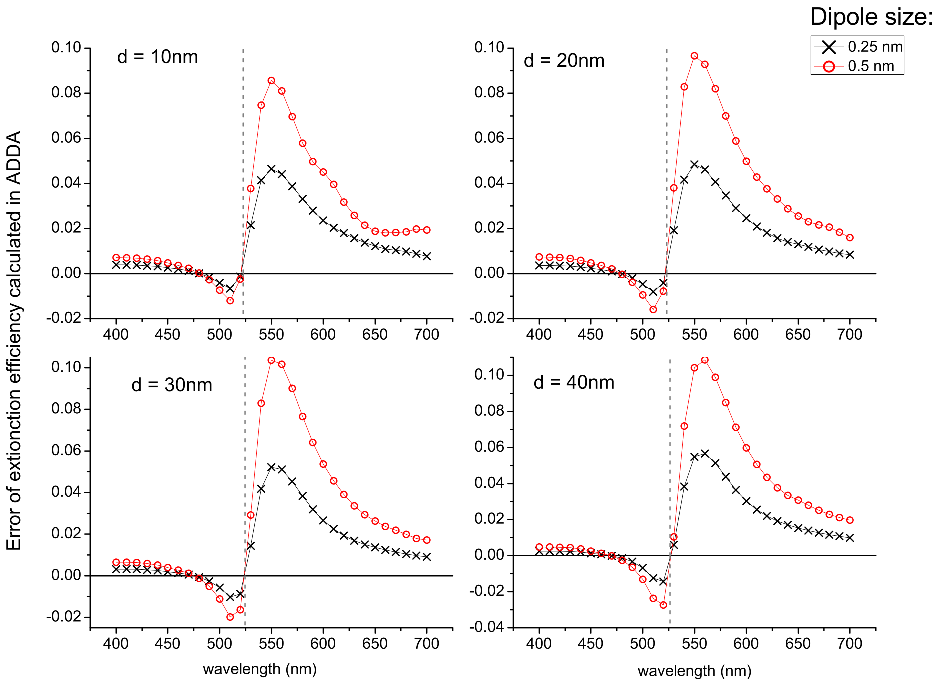

Figure S2: Differences between extinction efficiencies obtained from ADDA and Mie's solution ($Q_{ADDA} - Q_{Mie}$) for four spheres with diameter from 10 to 40 nm. For calculations in ADDA there were applied two dipole sizes - 0.25 and 0.5 nm. Dashed lines point resonance wavelength.

Generally, spectra calculated in ADDA are red-shifted and slightly overestimated in comparison to Mie's solutions. However, we are interested in changes of spectra, hence an impact of this bias should cancel out.

Although for large particles values of errors are greater we decided to use 0.5 nm dipoles because of limited amount of RAM memory. Application of smaller dipoles (0.25 nm) is required in case of smaller particles to avoid errors produced by surface's distortion, like artifacts in an enhancement of electric field.

1.2 Lorentz's profile fitting

Lorentz profile is fitted to the results from ADDA using a least squares method. Because changes in spectra are small, standard deviation of fitted parameters is a crucial issue.

As the accuracy of DDA is dependent on "dipoles per wavelength" parameter, a spectrum calculated for constant dipole size in a wide range (fig. S3a) is deformed. Therefore we tried to minimize this deformation by computing mainly in resonance's vicinity (fig. S3b). This strategy allows us to minimize standard deviation of fitted parameters and improve comparison of spectra. Specifically, figure S4. shows on an example of the same fits that resonance wavelength and maximum cross section are determined less accurate in case of wide range of wavelengths. Moreover, we verified that it is necessary to compute for the same wavelengths in case of a capsule without and with a protein.

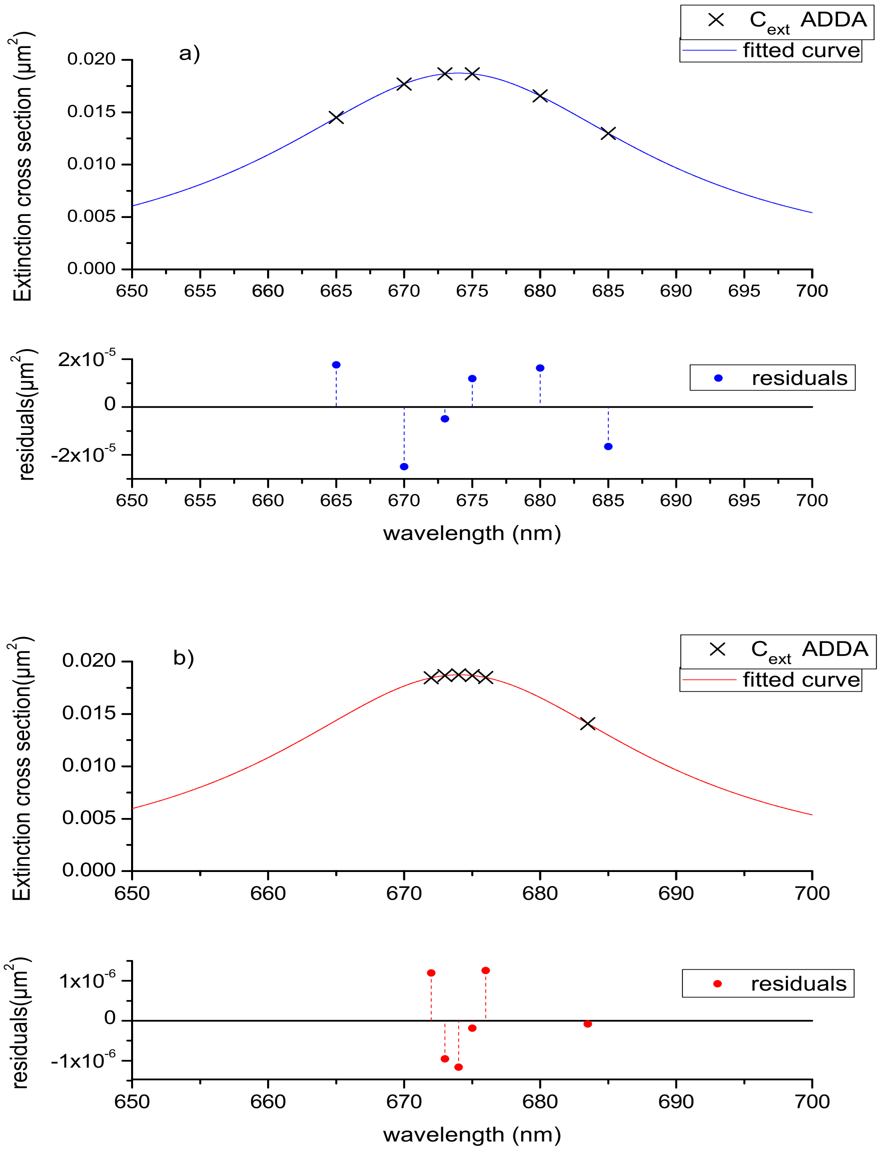

Figure S3: Calculated values of extinction cross sections for gold nanorod (diameter 20 nm and length 50 nm), fitted Lorentz profile and residuals.
(a) Wavelengths in a wide range, (b) wavelengths near resonance.
In the second case residuals are an order of magnitude less.

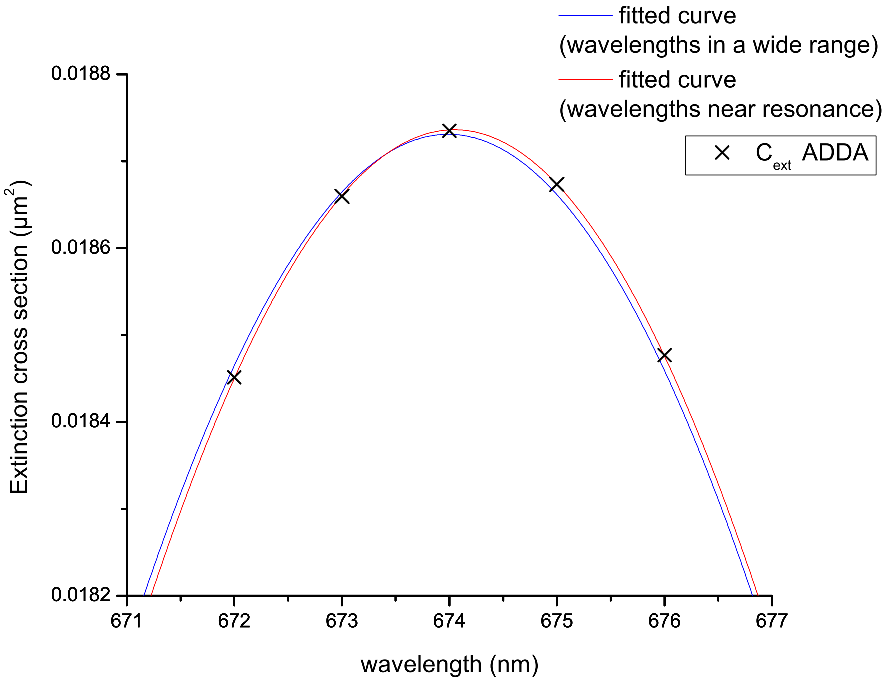

Figure S4: Close-up of the same fitted Lorentz's profiles: (blue line) - based on results in a wide range; (red line) - based on results in resonance's vicinity. Marked extinction cross sections calculated by ADDA allow for comparison of accuracy of both fits.

2 Width and maximum value of resonances, signal-to-noise ratio

In this section we present more results of our computations. Figure S5. shows maximum values of cross section and resonances FWHM (full width at half maximum) for scattering and absorption. In figure S6. SNR (signal-to-noise ratio) for scattering is presented. All calculations were made for a single gold nanorod with shell, placed in water, on glass substrate.

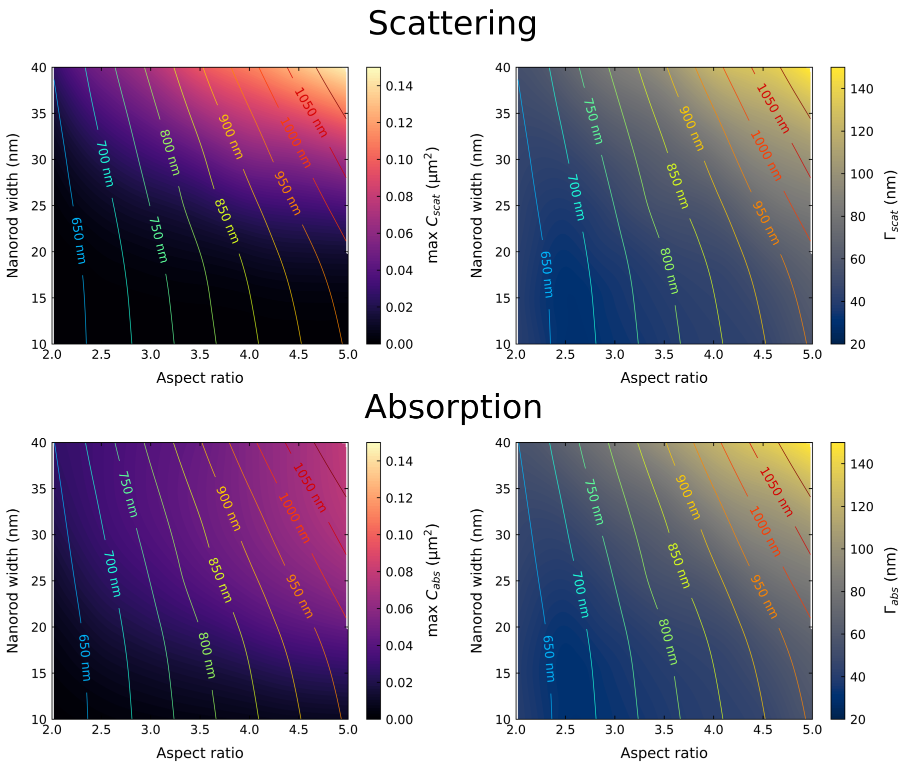

Figure S5: Maximum cross sections (a and c) and FWHM (b and d) for scattering and absorption of single gold nanorods. Nanorods' width ranging from 10 to 40 nm and aspect ratio ranging from 2 to 5. Contours show the LSPR resonance wavelength.

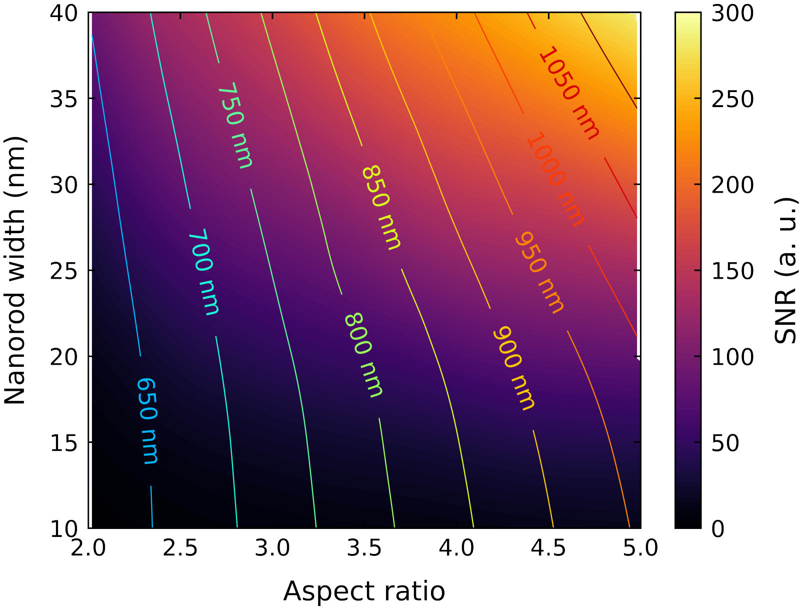

Figure S6: Signal-to-noise ratio for scattering of single gold nanorods. Nanorods' width ranging from 10 to 40 nm and aspect ratio ranging from 2 to 5. Contours show the LSPR resonance wavelength.

3 Impact of a non-ideal detector

For a non-ideal experimental system, the spectral characteristics of detectors QE would be the major factor influencing the obtained values CNR because the spectral position and width of LSPR vary significantly with nanorods size. In accordance with the general derivation of CNR presented in section 2.3 of the main text, the CNR is proportional to $\sqrt{A_{DET}}$, which in this case equals to $\sqrt{QE}$. Therefore, the use of standard silicon CCD or CMOS sensors optimized for visible light would favor the nanorods with lower aspect ratios which have a resonance closer to the maximum QE of the detector. As such, the optimum size would shift significantly towards shorter nanorods. However, for a detector optimized for the near-infrared wavelength range, i.e. back-illumination deep-depleted CCD, the CNR values corrected for the detector reach their maxima at similar sizes as for an ideal detector, though the distribution is notably narrower due to the steep drop of QE above 900 nm. The example spectra of QE for the two types of detectors from Princeton Instruments CCD cameras are plotted in Figure S7. The values of CNR corrected accordingly for standard CCD and deep-depleted CCD are shown in Figures S8 and S9, respectively for spectral and

fixed-wavelength scattering intensity sensing. The optimized sizes of nanorods for an ideal detector and the two types of CCD detectors are summarized in the table S1.

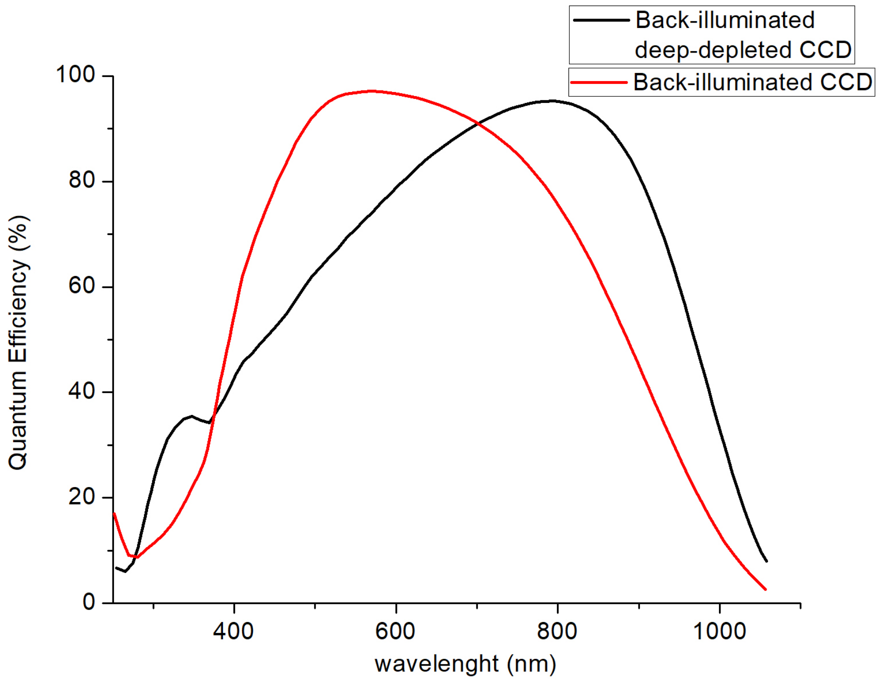

Figure S7: Example Quantum Efficiency (QE) curves of a back-illuminated CCD sensor, a back-illuminated deep-depleted CCD sensor from Princeton Instruments (https://www.princetoninstruments.com/learn/camera-fundamentals/quantum-efficiency)

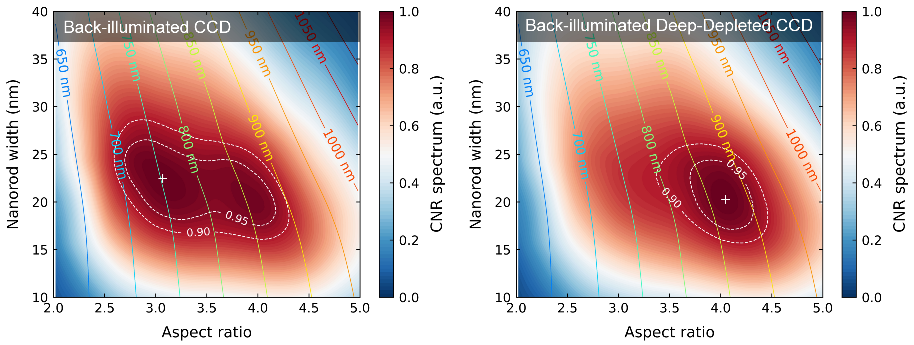

Figure S8: Contrast-to-noise ratio (CNR) for spectral sensing corrected for the detector's quantum efficiency of back-illuminated CCD and back-illuminated deep-depleted CCD, according to values plotted in Fig. S7

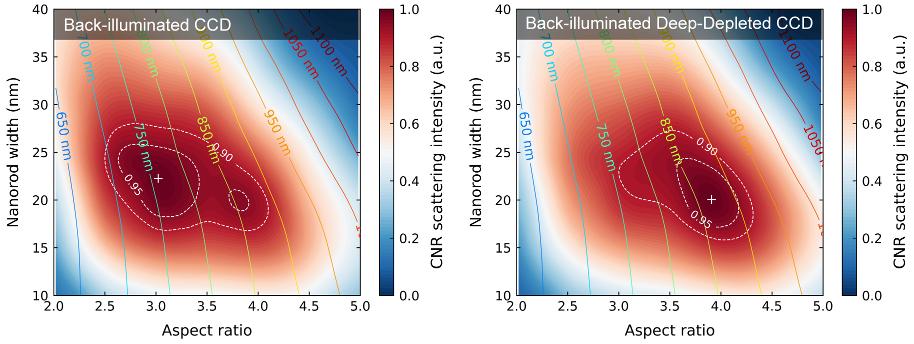

Figure S9: Contrast-to-noise ratio (CNR) for fixed-wavelength intensity sensing corrected for the detector's quantum efficiency of back-illuminated CCD and back-illuminated deep-depleted CCD, according to values plotted in Fig. S7

Table S1: Optimized sizes of nanorods for ideal detector and the two types of CCD detectors, back-illuminated CCD and back-illuminated beep-depleted CCD.

	Ideal detector	Back-illuminated CCD	Back-illuminated Deep-depleted CCD
Spectrum	21 × 87 nm	22 × 69 nm	20 × 82 nm
Fixed-wavelength scattering intensity	20 × 82 nm	22 × 67 nm	20 × 78 nm
